# Fecal microRNAs as Innovative Biomarkers of Intestinal Diseases and Effective Players in Host-Microbiome Interactions

**DOI:** 10.3390/cancers12082174

**Published:** 2020-08-05

**Authors:** Meysam Sarshar, Daniela Scribano, Cecilia Ambrosi, Anna Teresa Palamara, Andrea Masotti

**Affiliations:** 1 Department of Public Health and Infectious Diseases, Sapienza University of Rome, Laboratory Affiliated to Institute Pasteur Italia-Cenci Bolognetti Foundation, 00185 Rome, Italy; annateresa.palamara@uniroma1.it; 2 Research Laboratories, Bambino Gesù Children’s Hospital, IRCCS, 00146 Rome, Italy; andrea.masotti@opbg.net; 3 Microbiology Research Center (MRC), Pasteur Institute of Iran, 1316943551 Tehran, Iran; 4 Department of Public Health and Infectious Diseases, Sapienza University of Rome, 00185 Rome, Italy; daniela.scribano@uniroma1.it; 5 Dani Di Giò Foundation-Onlus, 00193 Rome, Italy; 6 IRCCS San Raffaele Pisana, Department of Human Sciences and Promotion of the Quality of Life, San Raffaele Roma Open University, 00166 Rome, Italy

**Keywords:** small non-coding RNAs, fecal microRNAs, bacterial small RNAs, extracellular vesicles, colorectal cancer, celiac disease, biomarkers, host-microbiome interplay

## Abstract

Over the past decade, short non-coding microRNAs (miRNAs), including circulating and fecal miRNAs have emerged as important modulators of various cellular processes by regulating the expression of target genes. Recent studies revealed the role of miRNAs as powerful biomarkers in disease diagnosis and for the development of innovative therapeutic applications in several human conditions, including intestinal diseases. In this review, we explored the literature and summarized the role of identified dysregulated fecal miRNAs in intestinal diseases, with particular focus on colorectal cancer (CRC) and celiac disease (CD). The aim of this review is to highlight one fascinating aspect of fecal miRNA function related to gut microbiota shaping and bacterial metabolism influencing. The role of miRNAs as “messenger” molecules for inter kingdom communications will be analyzed to highlight their role in the complex host-bacteria interactions. Moreover, whether fecal miRNAs could open up new perspectives to develop novel suitable biomarkers for disease detection and innovative therapeutic approaches to restore microbiota balance will be discussed.

## 1. Introduction

Communication molecules are necessary for “cohabitation” of bacterial and eukaryotic cells in the human body. These molecules are synthesized, released and internalized by both cell types in order to alter physiological cell function [1]. Communication occurs through small non-coding RNAs (sncRNAs), regulator molecules that can modulate gene expression. The sncRNAs include rRNAs, microRNAs (miRNA), tRNAs, small nucleolar RNAs (snoRNAs), small interfering RNAs (siRNAs) as well as piwi-associated RNAs (piRNAs) [2]. Bacterial and eukaryotic cells produce sncRNAs that are released by extracellular vehicles (EVs), a heterogeneous population of nano-sized cell-derived membranous vesicles originated from eukaryotic endosomal system (exosomes), plasma membrane (microvesicles: MVs), apoptotic bodies and/or from prokaryotic outer membrane vesicles (OMVs) or membrane vesicles (MVs) in Gram-negative and Gram-positive bacteria, respectively [3,4,5,6,7,8]. Biologically active components of EVs (termed “cargo”) are proteins, lipids, DNA, metabolites and extremely various RNA species (e.g., mRNAs, miRNAs, tRNAs) [9,10,11,12]. It is known that bacteria express a wide array of sncRNAs, approximately 50–400 nt in length, known as microRNA-size small RNAs (msRNAs) or generally bacterial-derived small RNA (bsRNAs) that are principally involved in the regulation of several physiological processes inside the bacterial cell [13,14]. Although poorly described so far, bsRNAs could regulate the expression of their respective target human genes [15]. In contrast, eukaryotic miRNAs are the most studied classes of sncRNA, regulating gene expression at the post-transcriptional level by binding to complementary sequences in 3′-untranslated regions (3′-UTRs) of target messenger RNAs (mRNA) [16,17,18]. It has been estimated that more than 60% of human protein-coding genes harbor predicted targets of miRNAs [19], and more than 30% of human genes have conserved miRNA binding sites in their 3′UTR [20]. Since such binding is not entirely homologous, miRNAs can act upon mRNA targets with limited complementarity; subsequently, a single miRNA can target a wide range of mRNAs as, in retrospect, multiple miRNAs can have similar mRNA targets as well [17,21,22,23,24]. Additionally, more than 90% of human Kyoto Encyclopedia of Genes and Genomes (KEGG) pathways contain genes that are targeted by at least one miRNA [19,25]. The partial or full complementarity of a miRNA with the mRNA target regulates the expression of target genes generally in a negative manner causing their degradation or translational inhibition [26,27]. Therefore, an upregulation of a specific miRNA generally leads to a lower expression of the protein encoded by its mRNA target, whereas the downregulation of a specific miRNA could lead to higher protein levels [27,28,29,30]. Eukaryotic miRNAs are found in several body sites and fluids since they are secreted by most cells under both physiological and pathological conditions. Circulating miRNAs can be found in the blood, saliva, urine, breast milk as well as in feces [31,32,33,34,35,36]. Moreover, circulating miRNAs were found to be differentially expressed in several human diseases. These findings began to make us understand how cells use miRNAs as communication molecules in the sophisticated dialogue among them. Due to their modulating function, circulating and particularly fecal miRNAs have emerged as a powerful tool for disease diagnosis and the development of miRNA-based therapeutic strategies. Focusing on colorectal cancer (CRC) and celiac disease (CD), herein we summarize the most important deregulated miRNAs and fecal-derived miRNAs associated to these intestinal diseases with the contribution of intestinal microbiota. The aim of this review is to report one fascinating aspect of miRNA function related to gut microbiota shaping and bacterial metabolism influencing. The role of miRNAs as “messenger” molecules for inter kingdom communications will also be analyzed to add novel elements in deciphering the complex host-bacteria interactions.

## 2. Biogenesis of miRNAs

**Figure 1:**
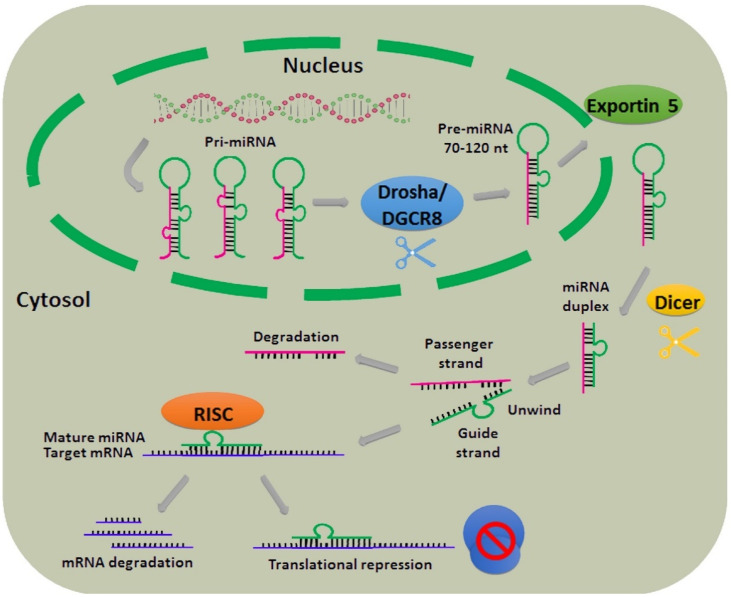
Biogenesis of miRNAs and their roles in translational repression.

Discovered in 1993, miRNAs are typically ∼18–22 nucleotides in length, single-stranded sncRNAs that are expressed by every cell type [37,38,39]. Unlike mRNAs, sncRNAs do not serve as templates for protein synthesis, but instead play a major role in modulating physiological responses through different mechanisms, such as RNA–RNA or RNA–protein interactions [40]. The biogenesis of miRNAs represents a series of sequential processes to generate mature miRNAs, as illustrated in Figure 1. In the canonical biogenesis pathway most miRNA genes are initially transcribed by RNA Polymerase II into a primary precursor (pri-miRNA, ≈200 nucleotides in length), and then processed in two steps catalyzed by members of the RNAse III family of enzymes, one nuclear and one cytoplasmic, named DROSHA and DICER, respectively. These pri-miRNAs are subsequently excised by the Microprocessor Multiprotein Complex (MMC), a dimer composed of RNAse III DROSHA enzyme and double-stranded RNA binding protein Pasha/DGCR8, into 60–70 nucleotide hairpin precursor miRNAs (pre-miRNAs). Hairpin precursors are exported from the nucleus to the cytoplasm by Exportin 5, where pre-miRNAs are additionally processed by DICER (RNase III endonuclease) into ~22 nucleotide-long mature duplex miRNA with a guide strand and a passenger strand. The guide strand associates with several RNA binding proteins, including argonaute 2 (Ago2), to form the microribonuclear protein (miRNP) complex known as the RNA-induced silencing complex (RISC). The mature miRNA may silence target gene expression via two mechanisms: binding to a target mRNA strand, thereby preventing its translation and/or promoting target mRNA degradation [41,42].

## 3. Clinical Applications of miRNAs

Recently, miRNAs have emerged as a new class of cellular molecules with potential diagnostic, prognostic and therapeutic implications. Up to now, the field of miRNA research has flourished with over 17,000 miRNAs discovered in 142 species. As of October 2018, more than 1917 precursors and 2654 human-derived miRNAs have been reported in miRBase 22.1 and it is estimated that these miRNAs regulate up to 50% of the transcriptome [43,44]. The key word “miRNA” pulls more than 85,600 publications from PubMed, and the first miRNA-targeted drug, Miravirsen (Santaris Pharma, Denmark), is currently in Phase II clinical trial. Miravirsen is an antisense oligonucleotide that inhibits both DROSHA- and DICER -mediated processing of miR-122 precursors which enhance hepatitis C virus (HCV) genome transcription [45,46,47,48]. The role of miRNAs in various cellular processes has been established, including cell division and death, cellular development, proliferation, replicative senescence, intracellular signaling and aging [16,23,49,50,51,52,53]. It is now clear that dysregulated expression of miRNAs can exert profound effects on cells function and, as a result, leads to various pathological and occasionally malignant outcomes [54,55,56,57]. The involvement of miRNAs was showed in more than 70 different diseases such as cancer, viral infection, gastrointestinal malignancies, diabetes, immune-related diseases, and neurodegenerative disorders [51,53,58,59,60,61,62,63,64]. In view of this, miRNAs are considered an interesting target for therapeutic intervention; to date, approximately 20 miRNA- and siRNA-based therapeutics are currently in the preclinical and/or in clinical trials [45,47].

### 3.1. Circulating miRNAs

An interesting class of miRNAs includes “circulating miRNAs” characterized by their active release through EVs or passive release upon cell death [12,30,65,66,67,68,69]. Several studies have shown that miRNAs can be identified in a variety of body fluids, including plasma, saliva, urine, seminal fluids, breast milk, cerebrospinal fluid and more recently also in feces, leading to the definition of circulating miRNAs [70,71,72,73,74]. Every miRNA has a unique nucleotide sequence and expression pattern in a certain cell type; however, although miRNAs show a high tissue specificity, body fluids can contain different or unique circulating miRNAs, that can putatively have several hundred gene targets, thereby generating an intricate picture [75]. Circulating miRNAs have been found associated with EVs, with high-density lipoproteins or with proteins involved in their processing such as the argonaute protein. EVs form includes microvesicles and exosomes that represent the common form of released extracellular miRNAs [30,65,68,69]. Nevertheless, miRNAs show the features of an ideal biomarker being available in body fluids, tissue- and disease-specificities, extremely stable and easily collected and detected using quantitative reverse transcription-polymerase chain reaction (qRT-PCR) [40,76,77,78,79]. The identification of circulating miRNAs opened the possibility to use them as biomarkers to understand their role in human health or diseases [80]. Although researchers are unveiling the miRNA functions, a lot of work should be done in characterizing circulating miRNAs including fecal miRNAs. Fecal miRNAs were first observed in 2008 [81], and several subsequent studies revealed that they are altered in many intestinal diseases, mainly in CRC [82,83,84]. Although fecal miRNAs are known to play a functional role in the intestine, their contribution in host-microbe communication is only beginning to be understood. More recently the role of bsRNAs started to be investigated highlighting new regulatory functions of intestinal bacteria on CRC [85].

The sophisticated miRNAs- and bsRNAs-mediated crosstalk between intestinal cells and microbiota is revealing new mechanisms by which eukaryotic and prokaryotic cells interact. In the following paragraphs we present a current overview about the state of art of fecal sncRNAs, principally human-derived, and their implication in intestinal homeostasis and diseases with a particular focus on miRNA interacting with bacterial cells.

### 3.2. Fecal miRNAs Contribute to Gut Microbiota Shaping

The intestinal microbiota has established itself in the scientific and clinic landscape as one of the most important components of the human body regulating its homeostasis. Moreover, a huge number of human diseases have been linked to changes in microbiota profiles underling the role of bacteria and their metabolites on host physiology [85,86,87,88,89]. Only recently, the interest has shifted to specific host components that are able to influence gut microbiota including host genetic factors and miRNAs [90,91,92,93].

The susceptibility of bacterial cells to eukaryotic miRNAs has been demonstrated by observing that some miRNAs such as miR-1, miR-130a, and miR-2392 can efficiently enter mitochondria and regulate the translation of specific mitochondrial genome-encoded transcripts [94,95,96]. These findings settled the bases for studying the impact of miRNAs on gut microbiota composition and dynamics [51,81,90,97,98,99]. Liu et al. for the first time demonstrated that host-fecal miRNAs can modulate gut microbiota composition by modifying the relative abundance of bacteria, considered at a family-level taxonomy. Indeed, mice defective in intestinal epithelial cell (IEC)-specific miRNA showed an increase of the diversity of bacterial genera compared to wild type mice. His pioneer study showed that fecal miRNAs are mostly secreted by IEC and by the homeodomain-only protein homeobox (HOPX)-positive cells, such as goblet and Paneth cells [100]. Moreover, most detectable fecal miRNAs are present in EVs form possibly because they are more stable. Using this knock-out mouse model, they observed a marked exacerbation of dextran sulfate sodium (DSS)-induced colitis in IEC-specific miRNA-deficient mice compared to wild type that was ameliorated by transplanting wild type fecal miRNAs. This observation suggests that bacterial growth may be controlled by extracellular fecal miRNAs and may prove the clinical relevance for their development for therapeutic applications in humans, as the efficacy and safeness of fecal microbiota transplant [101,102,103,104]. Interestingly, it was demonstrated that specific fecal miRNAs were able to enter *Fusobacterium nucleatum* and *Escherichia coli* cells, promoting their growth. In particular, human miR-515-5p increased the ratio of *F. nucleatum* 16S rRNA/23S rRNA transcripts as miR-1226-5p and miR-4747-3p increased the mRNA levels of *E. coli* yegH and RNaseP, respectively. Vice versa, miR-1224-5p and miR-663 reduced the mRNA levels of *E. coli* rutA and fucO, respectively [100]. YegH belongs to the flavin adenine dinucleotide binding proteins associated to the membranes and probably acts as a flavoprotein oxidoreductase enzyme; together with enhanced expression of RNaseP, YegH contributes to the increase of bacterial metabolism and growth. RutA is a pyrimidine monooxygenase involved in pyrimidine metabolism (degradation and biosynthesis) and FucO is a lactaldehyde reductase involved in carbohydrate metabolism. Although predicted, these functions can be profoundly altered by miRNA contributing to bacterial metabolism shift. Ji Y et al. demonstrated that differentially expressed miRNA, associated to intestinal bowel diseases (IBD), target the intestinal bacteria *F. nucleatum*, *E. coli* and the segmental filamentous bacteria (SFB) [105]. In particular, miR-199a-5p inhibited the proliferation of SFB but did not affect the in vitro multiplication of *F. nucleatum* and *E. coli*. On the contrary, miR-1226 and miR-515-5p inhibited the growth of *F. nucleatum* or *E. coli* and promoted SFB replication. Finally, miR-548ab inhibited the growth of *F. nucleatum* and *E. coli* having no effect on the growth of SFB [105]. These data indicate that the differential expression of fecal miRNAs affect the growth of intestinal bacteria. Moreover, miRNAs can fine tune the relative abundance of the different bacterial species. Hence, miRNAs became an important “influencer” of microbiota composition and dynamic. Taking into account that changes in microbiota composition are associated to several human diseases, acting on miRNAs could contribute to manipulate microbiota balance. Furthermore, the idea that microbes might actively and selectively take up different miRNAs and that these miRNAs in turn affect microbial growth supports the hypothesis of an inter kingdom communication via miRNAs. *F. nucleatum* and *E. coli* have been previously reported to drive CRC [100,106]. Nucleic acids from both bacteria were found to co-localize with host miRNAs, possibly leading to changes in bacterial replication rates. Identifying host-derived miRNAs targeting bacterial gene expression would be of paramount importance to study the relationship at the bacterial-host interface. Recently, Teng et al. demonstrated that miRNAs encapsulated in plant-derived exosome-like nanoparticles (ELNs) can enter bacteria specifically modifying their growth rate [99]. Indeed, mice fed with Ginger-derived ELNs (GELNs) showed a different microbiota profile compared to mice treated with Phosphate Buffered Saline (PBS), with an increase of *Lactobacillaceae* and *Bacteroidales* and a decrease of *Clostridiales*. A deeper analysis demonstrated that GELNs induce the growth of several species belonging to *Lactobacillus* and that GELN lipid composition mediates their specific uptake by *Lactobacillus rhamnosus* (LGG). RNA sequencing and proteomic data analyses indicated that 398 mRNAs and 149 proteins were increased in GELN-treated LGG; vice versa, 249 LGG mRNAs and 133 proteins were downregulated [99]. The alignment of miRNA nucleotide sequences with bacterial mRNAs revealed that the GELN gma-miR396e targets the LexA mRNA, and its downregulation was directly linked to the LGG faster growth. Moreover, GELN mdo-miR7267-3p downregulates the expression of the monooxygenase *ycnE* gene resulting in a bacterial metabolic change demonstrated by the accumulation of tryptophan and the reduction of its derivative metabolite indol-3-acetamide. Finally, GELN miR167a-5p downregulates the LGG pilus-specific *spaC* gene [99]. Altogether, these results definitively demonstrate that eukaryotic-derived miRNAs manipulate bacterial gene expression impacting on bacterial metabolism and phenotypes such as the motility. We are just at the beginning to identify miRNAs targeting bacteria and to understand their biological function, including microbiota modulation. Vice versa, a more detailed scenario is available about the impact of microbiota on intestinal-derived miRNAs. The most important discoveries are extensively reviewed elsewhere and briefly summarized below [26,92,107].

**Figure 2:**
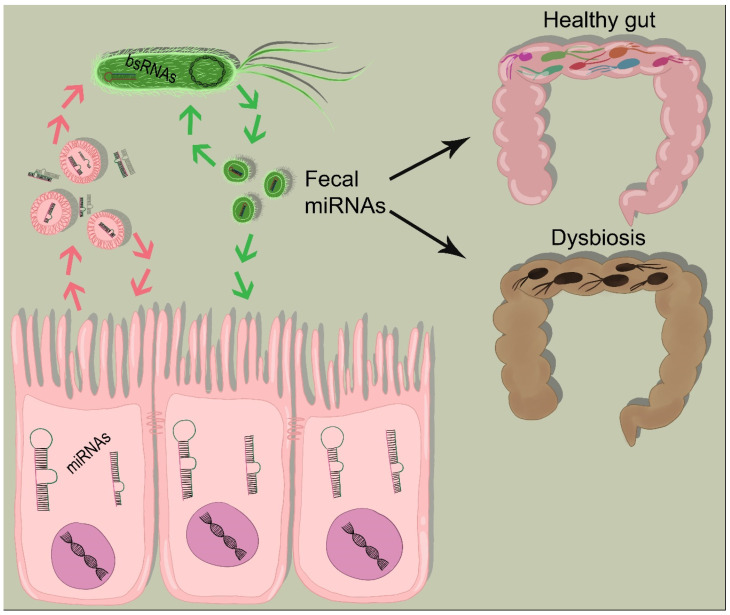
Molecules as messenger in bacteria-host communication. Fecal miRNAs from eukaryotic cells and bsRNAs from bacteria can be taken up by bacteria or by host cells, respectively, and critically mediate bacterial–host communication. Host-derived miRNAs can regulate bacterial gene transcripts and affect bacterial growth which might promote intestinal dysbiosis.

Dalmasso and colleagues demonstrated for the first time that microbiota, by regulating miRNAs, alters host gene expression [51]. The authors showed that miRNA expression profiles of germ-free (GF) mice were markedly different from GF mice colonized with the microbiota from pathogen-free (SPF) mice both in ileum and colon. Furthermore, they observed that differentially expressed miRNA such as miR-665, which was found downregulated in colonized GF mice, targeted the Abcc3 gene (an ATP-binding cassette transporter) in the colon [51]. Using the same approach, Singh et al. showed that different levels of miRNA expression impact on the regulation of the intestinal barrier function and homeostasis [80]. It was also shown that commensal bacteria induce the expression of miR-21-5p in IECs upregulating the ADP ribosylation factor 4 (ARF4) which, in turn, alters tight junction expression [108]. Viennois et al. showed that fecal miRNA profiles are strictly dependent on the gut microbiota. Indeed, colitogenic mice are characterized by a fecal miRNA’s profile that became a reliable inflammatory “signature” [84]. Interestingly, the foodborne pathogen *Listeria monocytogenes* modifies microbiota-regulated miRNA profiles upon infection [109]. Furthermore, putative targets of the differentially expressed miRNAs are associated to the immune response. Hence, gut microbiota by modulating host miRNAs contributes to reprogramming host transcriptional landscape during infections [109]. IECs are the major source of intestinal miRNAs but, at the same time, they are the target of miRNA regulatory function. It was recently showed that miRNA profiles are very different across the IEC subtypes (e.g., goblet, enteroendocrine, tuft and Paneth cells). Moreover, miRNA sensitivity to microbial status is highly cell type-specific, suggesting a new mechanism by which microbiota can interfere with a specific cell type behavior [110]. The gut ecosystem is an extreme complicated environment in which eukaryotic and prokaryotic cells cohabit and interact by using several “informative” molecules such as sncRNAs. Deciphering this communicative strategy will extend the knowledge on miRNAs biologic role and will help in defining lines of intervention for human health (Figure 2).

### 3.3. Fecal miRNAs as Potential Biomarkers to Link Colorectal Cancer (CRC) and Gut Microbiome

CRC is still one of the three most aggressive cancers which leads to one million new cases every year with an increasing incidence globally [111,112]. CRC is commonly regarded as a multistep process, initially from aberrant crypt foci, through benign precancerous lesions (adenomas), followed by malignant tumors (adenocarcinomas) over an extended period [113]. Screening and early diagnosis of cancer are the main approaches for CRC prevention; at present, the common used tests for CRC are colonoscopy, computed tomography (CT) colonography (virtual colonoscopy), multitargeted stool DNA test, detection of serum carcinoembryonic antigens (CEA), detection of carbohydrate antigen 19-9 (CA19-9) and a fecal immunochemical test (FIT) [114,115]. Indeed, fecal specimens contain exfoliated tumor cells and several tumor markers that are already used for CRC screening as well as stable miRNAs [81,116,117,118,119,120,121]. Among these available tests, colonoscopy screening is the most common method for detection of CRC [122]. However, colonoscopy is an invasive operation, expensive and it has been estimated that 25% of polyps are not detectable during the screening [122,123,124]. Moreover, the other available tests have several limitations such as low specificity and sensitivity [125]. In recent years, the identification of novel CRC biomarkers has become one of the main challenges of cancer research which highlights the importance of developing effective, cost-efficient, non-invasive tests for CRC screening. The possibility to explore intestinal-derived miRNAs as putative indicators of CRC opened new research lines focused on miRNA profiling in CRC patients versus healthy controls. Michael et al. in 2003 provided the first evidence of differentially regulated miRNAs from CRC and normal mucosa specimens resected from the same patient [126]. As outlined before, the diagnostic value of circulating miRNAs for CRC detection was suggested in several studies [81,127,128,129,130,131,132,133], and a growing body of evidence is highlighting fecal miRNAs as powerful clinical tools [81,82]. Ahmed et al. demonstrated the feasibility of fecal RNAs isolation and miRNAs quantification in stool samples by detecting specifically downregulated miRNAs in CRC patients compared to healthy individuals [81]. After the establishment of isolation and quantification protocols, Ahmed et al. carried out a global microarray expression studies on stool samples to evaluate the expression of miRNAs in patients exhibiting various stages of CRC progression (tumor-lymph node-metastasis (TNM) stages 0-IV). Among 212 differently expressed miRNAs, they identified 20 miRNAs (12 downregulated and 8 upregulated) discriminating not only CRC patients from healthy controls but also different TNM stages with high sensitivity and specificity (Table 1) [134]. Based on this, Wu et al. observed the upregulation of miR-21 and miR-92a in CRC bioptic specimens compared to adjacent normal tissues and confirmed this trend analyzing stool samples from CRC patients and healthy controls (Table 1). In addition, stool-derived miR-92a, but not miR-21, was significantly higher in patients with polyps than in controls suggesting a stage-specific miRNA pattern. To test the potential diagnostic application, these authors assayed the sensitivity of miR-92a in detecting CRC patients. Results showed that miR-92a had a sensitivity of 71.6% and 56.1% for CRC and polyp, respectively, and a specificity of 73.3%. Finally, they showed that removal of tumor resulted in reduced stool miR-21 and miR-92a levels, and removal of advanced adenoma resulted in the decrease of the stool miR-92a level [135].

Downregulation of overexpressed tumoral miRNAs after curative surgery was also observed by Rotelli et al., suggesting that a possible modulation of these miRNAs can represent a therapeutic approach [83]. More recently, Yau et al., in a systematic analysis of fecal-based miRNAs, showed that fecal miR-21, miR-92a and their combination are promising non-invasive biomarkers for fecal-based CRC screening [121]. Importantly, a meta-analysis of over 500 CRC patients reported that the levels of miR-92a in the blood can be detected with a sensitivity and specificity of 76% and 64%, respectively. This result highlights the important role of this circulating miRNA for CRC diagnosis [136]. Among those fecal miRNAs suggested as biomarkers for CRC screening, the miR-17-92 cluster (miR-17, miR-18a, miR-19a, miR-19b, miR-20a, miR-92a) and miR-135 were reported to have a good sensitivity and specificity (74.1% and 79.0%, respectively) [137]. Likewise, the expressions of miR-21, miR-146a, miR-221 and miR-18a were found to be upregulated in CRC tissue compared to adjacent normal tissue [138,139]. Accordingly, these miRNAs were found to be overexpressed also in fecal samples, corroborating their CRC-predictor function (Table 1) [138]. Furthermore, specific fecal miRNAs, such as miRNA-29, were found to be upregulated in rectum cancer and downregulated in colon cancer, suggesting the feasibility of using differential miRNA expression patterns as cancer fingerprints [140]. Importantly, dysregulated miRNAs are abundantly present in stools that contain exfoliated colonocytes and blood as a consequence of the disease [81,134,135,141,142,143]. Analyses on stool-based miRNAs were shown to be reproducible due to their high stability in feces. Recently, a systematic analysis conducted on 51 dysregulated fecal miRNAs showed the potentiality of individual or multiple stool-based-miRNAs as non-invasive CRC biomarkers [144]. Despite their valuable role as biomarkers, further investigations on larger study populations of CRC patients are required to enhance the sensitivity and specificity for cancer diagnosis.

One of the most studied topics related to CRC is the contribution of gut microbiota on cancer development and progression. Bacterial relative abundance and composition have an impact on carcinogenesis. Dysbiosis is characterized by the expansion and depletion of bacterial species; in CRC, it has been demonstrated the expansion of specific bacterial pro-tumorigenic species, such as *F. nucleatum*, *E. coli* and *Bacteroides fragilis,* and the reduction of species that could have protective and beneficial roles against the overgrowth of tumor-associated bacteria [145]. Moreover, through the recent use of fecal shotgun metagenomics and machine learning models, it was shown that CRC is characterized by different levels of microbiota variability ranging from species profiles to gene variants in one single bacterial strain [146,147]. Indeed, these datasets or classifiers include CRC-associated bacterial species and functional genes possibly involved in eukaryotic cell toxicity and tumorigenesis [146,147]. The building of these datasets allows a more reliable comparison among data from different studies to detect valid microbiota-derived CRC biomarkers. The first investigation on the association between miRNA expression and microbiota composition in human CRC was performed by Yuan and colleagues in 2018 [148]. The miRNA and microbiome profiles in CRC specimens were compared to adjacent normal tissues collected from CRC patients. They identified 76 differentially expressed miRNAs, including the known oncogenic miRNAs miR-182, miR-503 and mir-17~92 cluster. By analyzing the microbiota profiles, they showed that the relative abundances of specific bacterial taxa and genera correlated to differentially expressed miRNAs [148]. For example, the genus *Blautia* is abundant in normal tissues, whereas it is poorly represented in tumor samples; interestingly, *Blautia* is positively correlated with the expression level of miR-139, highly expressed in normal tissue, and negatively correlated with miR-20a, miR-21, miR-96, miR-182, miR-183 and miR-7974, all overexpressed in tumor tissues. Searching for putative targets of deregulated miRNAs in KEGG bacterial database revealed a positive correlation with bacterial pathways including transporters, peptidoglycan, and terpenoid backbone biosynthesis [148]. Altogether, these results suggested a possible way by which miRNAs modify the microbiota composition and bacterial metabolic function, as previously described. On the other hand, microbiota is able to influence host miRNA profiles indicating a bidirectional miRNA-mediated interaction between host and bacteria. Very recently Tomkovich and colleagues were able to unveil a complex interaction among tumorigenic bacterial community, deregulated host-derived miRNAs and CRC development. Germ-free Apc^MinΔ850/+^;Il10^-/-^ mice, representing a model for CRC, were transplanted with tumorigenic bacterial community isolated from biofilm-positive and –negative tissues from human CRC tumor and healthy mucosa, respectively, being biofilms a condition associated with adenomatous lesions and CRC progression [88,149,150]. By miRNA sequencing on stools, they found deregulated fecal miRNAs in Apc^MinΔ850/+^; Il10^-/-^ mice associated with tumorigenic bacterial community compared to mice associated with non-tumorigenic bacteria, as expected. Moreover, the human homologous miRNAs (hsa-miR-21-5p, hsa-miR-142-5p, and hsa-miR- 146a-5p) were shown to be increased in CRC patients highlighting the impact of tumorigenic bacteria on host-derived miRNAs [150]. Interestingly, differentially regulated miRNAs (miR-2137, miR-5126, miR-6239, miR-6240 and miR-6538) were predicted to target bacterial genes, including genes regulating motility, secretion, outer membrane proteins, stress response, iron acquisition and carbohydrate utilization/transport. Furthermore, they observed that several genes belonging to these functional categories were upregulated in Apc^MinΔ850/+^; Il10^-/-^mice associated with tumorigenic bacterial community [150].

Host-bacterial communication is also mediated by bsRNAs. The study of bsRNAs associated to human diseases is an extremely recent breakthrough in the field of sncRNAs. An interesting point of view was presented by Tarallo and colleagues who highlighted a CRC signature composed of profiles of miRNAs, bsRNAs, and microbiota. By combining transcriptomic and metagenomic data from stool samples, they showed that bsRNA profiles reflect the differences in microbial profiles of healthy subjects vs. patients with adenomas and CRC [85]. For example, the bsRNA 6S RNA was found to be overexpressed in CRC patients; this bsRNA correlated with the regulation of bacterial stationary phase promoting *E. coli* survival under nutritional limitation. Furthermore, the overexpression of ryfD, ffs, and FnrS bsRNAs regulate *E. coli* biofilm, swarming ability, signal recognition particle system and cell metabolism in anaerobic growth conditions. Overall, a differential regulation of specific *E. coli* bsRNAs could reflect the selection of *E. coli* strains more adapted to colonize the tumor microenvironment which share phenotypic traits [85,88]. Although being interesting, the contribution of bsRNAs on human diseases is poorly investigated; vice versa, a lot of attention is focused on human-derived miRNAs and how they can alter bacterial species profiling and their metabolism.

Overall, the combination between miRNA and bsRNAs sequencing and 16S rDNA profiling represents a powerful approach to deepen the knowledge about sncRNAs regulating bacterial behavior within tumor environment. In addition, it could reveal novel sncRNAs suitable for CRC diagnosis but also for microbial modulation in view of therapeutic applications. Overall, fecal sncRNAs represent undoubtedly a promising CRC biomarker; however, studies available on patients are exploratory, include a low number of subjects and employ different and not-standardized methodologies. Moreover, extended analyses on fecal sncRNAs modulation and their impact on gene expression are mostly predictive and performed using animal or in vitro models. Hence, the diagnostic power of this biomarker has not been fully demonstrated yet. To this end, the most relevant fecal human-derived miRNAs proposed as CRC biomarkers are listed in Table 1 in order to depict the hypothetical miRNA panel to be considered for clinical researches.

### 3.4. Can Fecal miRNAs Be Celiac Disease Biomarkers Interacting with Gut Microbiota?

CD is a genetic autoimmune disorder mainly affecting the small intestine, elicited by an aberrant inflammatory response to dietary gluten proteins found in wheat, rye and barley [27,162,163,164]. The complex interaction between genetic and environmental factors characterizing CD has not been completely elucidated yet. There is a growing body of evidence that exposure to gliadin induces an increased zonulin release which determines the opening of the tight junctions, thereby affecting intestinal barrier integrity. This, in turn, enhances the inflammatory response that contributes to CD pathogenesis and disease progression [165,166].

Substantial efforts have been made to identify novel molecular biomarkers for the diagnosis of CD or for the follow up of intestinal damage progression [167,168,169,170,171,172]. Among them, miRNAs have recently been studied. Indeed, the comparison between miRNA profile extracted from bioptic tissues of CD and control children revealed that the 20% of the tested miRNAs were differently expressed [167]. The NOTCH signaling pathway regulates the development of the intestine and its homeostasis. This pathway includes the NOTCH1 receptor and the Krüppel-like transcription factor 4 (KLF4). Interestingly, it was reported that the upregulation of miR-449a reduced NOTCH1 and KLF4 in HEK293 cell culture model. Accordingly, NOTCH1 and KLF4 were decreased in the small intestine of children with active CD and on a gluten-free diet (GFD) compared to controls, suggesting that deregulated miRNAs characterize CD disease [167]. The biological consequence of deregulated NOTCH signaling pathway consists into changes in the composition of intestinal tissue by increasing proliferative undifferentiated cells and reducing mature goblet cells, altering the homeostasis of the intestinal mucosal environment [167]. Another study evaluated differentially expressed miRNAs isolated from duodenal mucosa from adult CD patients and non-CD subjects. What was observed was the deregulation of several miRNAs, including miR-31-5p, miR-192-3p, miR-194-5p, miR-551a, miR-551b-5p, miR-638 and miR-1290 [169]. Noteworthily, miR-192-3p levels were subjected to a specific modulation by gliadin peptides and the miRNA cluster miR-192/194 was shown to be involved in matrix remodeling, possibly leading to cell apoptosis which, in turn, promotes the proliferative state of intestinal crypts. Magni et al. achieved similar results by analyzing differentially expressed miRNAs in the duodenum of adult CD patients compared to controls. In particular, seven miRNAs were significantly downregulated in CD and the in-silico analysis revealed possible gene targets involved in innate and adaptive immunity [168]. Due to their downregulation, the expression levels of inflammatory genes could increase, worsening clinical conditions. Moreover, miR-192-5p and miR-31-5p expressions were triggered by gliadin exposure in CD patients, as shown also by Vaira and colleagues [168,169].

These studies opened the possibility to explore miRNAs as markers of CD. Accordingly, several papers described circulating miRNA as a powerful tool to detect and to follow up celiac patients, as already shown for CRC. Indeed, detailed knowledge on the expression levels and possible roles of circulating miRNA in CD are available and extensively reviewed elsewhere [170,171,172,173].

As outlined for CRC, gut microbiota alteration is also associated to CD; bacterial composition shift can be the cause or can contribute to the onset and clinical manifestations of CD [173,174,175,176,177,178,179,180,181,182,183,184,185,186,187,188]. An overall unbalance between Gram-positive and Gram-negative bacteria is frequently observed in CD patients resulting in intestinal dysbiosis [163,174,175,176]. In particular, an increase in members belonging to *Bacteroides, Firmicutes, Enterobacteriaceae* and *Staphylococcus*, and a decrease in *Bifidobacterium*, *Streptococcus*, *Prevotella* and *Lactobacillus* spp. were observed in duodenal specimens from CD patients compared to normal controls. Accordingly, analyses of fecal samples showed an increase in *Bacteroides, Clostridium leptum, Histolitycum, Eubacterium* and *Atopobium* and a decrease in *Bifidobacterium* spp., *B. longum, Lactobacillus* spp. and *Leuconostoc* compared to the normal population [163,175,177,178,179,180,181,182,183,184,185]. The overall composition and dynamic of gut microbiota in CD patients were reviewed elsewhere [165,173,174,175,176,185,186]. Although there are ecological differences in the upper and lower part of the intestinal tract that influence the microbiota composition, the bacterial signatures detected in biopsies and feces of CD patients were found to be correlated [179,188]. This is indicative that the fecal microbiota partly reflects the ones associated to the small intestine, thereby implying that fecal samples might have a diagnostic value suitable for pathogenesis evaluation. Differently from CRC, scarce information is available on miRNAs deregulated by microbiota in CD patients and even less is known about miRNAs targeting bacteria. This miRNA-mediated crosstalk is a totally uninvestigated research field in the context of CD. To fill this gap, the study of Mohan et al. combined the microbiota dysbiosis with the expression levels of selected miRNAs. Firstly, they showed that in a model of gluten-sensitive (GS) macaques under gluten-containing diet (GD) the diversity of gut microbiota was significantly lower with respect to the healthy, age-matched peers. This phenotype was restored by the GFD indicating a direct relationship between microbiota composition and tissue damages mediated by inflammatory response to gluten. The analysis of miRNAs revealed several upregulated miRNAs, such as miR-203, miR-204, miR-23b and miR-29b. Interestingly, the analysis of putative miRNA targets highlighted their complementarity on 16S ribosomal RNA of bacterial species such as *Lactobacillus reuteri*, *Prevotella stercorea* and *Streptococcus luteciae* that were found to be overrepresented in the fecal sample of GS macaques under GD [173]. To the best of our knowledge, this is the only study highlighting the role of miRNAs potentially targeting bacteria and then contributing to dysbiosis in CD. In this context, fecal miRNAs represent ideal candidates to study intestinal diseases and gut microbiota shaping. However, very few data are available on fecal miRNAs up to date, revealing a hole in the scientific literature. Fecal samples are highly informative for intestinal related diseases and extremely easy to collect without any invasive procedures. These characteristics make fecal miRNAs ideal biomarkers for several gut disorders and, therefore, should be deeply investigated.

## 4. Conclusions and Future Perspectives

Cumulative evidence has outlined the aberrant expressions of miRNAs and their roles in intestinal diseases such as CRC and CD. Eukaryotic-derived miRNAs for disease diagnosis, monitoring and treating are highly promising tools as demonstrated by several ongoing clinical miRNA-based trials. On the other hand, we have extended our knowledge on microbiota structure and modification associated to human health and diseases. Recently, the impact of microbiota in eukaryotic miRNA synthesis and regulation has emerged showing another mechanism through which bacteria affect human homeostasis. In the context of “omic” studies, the combined identification of bacterial species, functional genes as well as sncRNA profiling unveils a possible way to define a microbiota “signature”. Indeed, host-derived miRNAs are specifically taken up by bacteria resulting in bacterial metabolic and proliferation rate changes. Hence, miRNAs influence microbiota composition dynamic and expression, possibly regulating the eu- and dys-biosis. The existence of miRNA-based communication at the host–microbiota interface could provide new insights for the diagnosis, staging and monitoring of intestinal diseases. Based on this, fecal miRNAs offer the possibility to have a promising biomarker for disease diagnosis and treating. However, several gaps should be filled on this new opened research field. Fecal miRNAs associated to CD are poorly characterized, and detailed studies are required to identify and verify this class of miRNAs as biomarkers for clinical use, as for circulating miRNAs. Indeed, the few studies available addressing fecal miRNAs demonstrated their role in affecting bacterial behavior and their impact, but only in in vitro models. Thus, we ought to collect much more data from basic researches to unravel the link(s) between miRNA expression levels and microbiota profiling. By exploring the most innovative technologies such as high-throughput omic approaches, human-derived organoids to unravel microbiota-host interaction network, we will reach fundamental knowledge about miRNAs’ regulatory functions for their validation in clinical practice.

## Figures and Tables

**Table 1:** Fecal miRNAs as diagnostic biomarkers for colorectal cancer (CRC).

Fecal miRNAs	Expression Level CRC VS. HC	Ref
miR-17-92 cluster *	Up	[137]
miR-20a	Up	[151]
miR-21	Up	[82,135,139,152,153,154,155]
miR-135	Up	[118,137]
miR-144	Up	[155,156]
miRNA-146a	Down, although not significant	[139]
miR-29a	Down	[140]
miR-223	Up	[156]
miR-223	Down	[140]
miR-34a	methylation	[157]
miR-34b/c	methylation	[157]
miR-221	Up	[138]
miR-92a	Up	[135,139,153,154,155,158]
miR-224	Down	[140]
miR-106a	Up	[81,82,153]
miR-106b	Up	[82]
miR-143	Down	[159]
miR-145	Down	[159]
miR-4478	Down	[160]
miR-135b	Up	[118]
miR-1295b-3p	Down	[160]
3 types miRNAs	Up-regulated miR421, miR130b-3p, and miR27a-3p	[161]
14 types miRNAs	Up-regulated miR-21, -106a, -96, -203, -20a, -326 -92 Down-regulated miR-320, -126, -143, -484-5p, -16, -145 -125b	[81]
20 types miRNAs	Up-regulated miR-7, miR-17, miR-20a, miR-21, miR-92a, miR-96, miR-106a, miR-134, miR-183, miR-196a, miR-199a-3p, miR-214Down-regulated miR-9, miR-29b, miR-127-5p, miR-138, miR-143, miR-146a, miR-222, miR-938	[134]

Up and Down indicate Up-regulated and Down-regulated miRNA expression in a population with CRC compared with healthy volunteers, respectively. CRC: Colorectal Cancer; miRNA: microRNA; HC: Healthy Control. * The miR-17-92 cluster includes miR-17, miR-18a, miR-19a, miR-20a, miR-19b-1 and miR-92a.
